# Injury Profiles in Korean Youth Soccer

**DOI:** 10.3390/ijerph17145125

**Published:** 2020-07-16

**Authors:** Inje Lee, Hee Seong Jeong, Sae Yong Lee

**Affiliations:** 1 Department of Physical Education, Yonsei University, Seoul 03722, Korea; indie90@yonsei.ac.kr; 2 International Olympic Committee Research Centre KOREA, Yonsei University, Seoul 03722, Korea; 3 Institute of Convergence Science, Yonsei University, Seoul 03722, Korea

**Keywords:** adolescent, epidemiology, injury surveillance, soccer, youth player

## Abstract

We aimed to analyze injury profiles and injury severity in Korean youth soccer players. Data on all injuries that occurred in U-15 youth soccer players during the 2019 season were collected from 681 players of 22 teams through a medical questionnaire. The questionnaire was based on injury surveillance procedures of the Federation International de Football Association Medical and Research Centre and International Olympic Committee, and it comprised questions on demographic characteristics, training conditions, and injury information. Among all players, defenders accounted for 33.0%, followed by attackers (30.7%), midfielders (26.8%), and goalkeepers (7.9%). Most players played soccer on artificial grounds (97.4%). Injuries occurred more frequently during training (56.3%) than during matches (43.7%). Recurrent injury rate was 4.4% and average days to return to full activities were 22.58. The ankle (26.6%) and knee joints (14.1%) were the most common injury locations, and ligament sprains (21.0%), contusions (15.6%), and fractures (13.9%) were the most frequent injury types. In conclusion, Korean youth soccer players have a high injury risk. Therefore, researchers and coaching staff need to consider these results as a key to prevent injuries in youth soccer players and injury prevention programs may help decrease injury rate by providing injury management.

## 1. Introduction

Soccer is one of the most popular team sports in the world [1]. In their 2007 report, the Federation International de Football Association (FIFA) [2] indicated that more than 200,000 professional and 265 million amateur players were registered worldwide. In Korea, there are 4508 male soccer teams and 153,085 registered players in the Korea Football Association (KFA) including 747 elite youth teams and 21,326 elite adolescent players [3,4]. However, the number of people who play soccer in Korea is high considering players who are not officially counted. According to a previous study [5], the incidence of injuries is higher in soccer than those in other team sports. Because the popularity of soccer has increased, many more Korean youth participate in soccer, which may result in increased incidence of injuries and the early retirement of youth soccer players. Therefore, epidemiological studies are needed to prevent injuries that affect an athletic career.

Based on the data from previous studies [1,6,7,8,9,10,11,12,13,14,15,16], the most common injury location in soccer players is the lower extremity, with a high proportion of injuries occurring in the ankle and knee joints. In addition, the most frequent types of injury in soccer players are sprains, strains, and contusions [6,9,10,15]. Many researchers [6,9,10,15] have investigated the differences in the incidence of injury between matches and training sessions and reported that the incidence of injuries is higher during matches than during training sessions. Furthermore, two review papers [10,16] compared the incidence of injury among youth soccer players with that among professional players and found conflicting results. Wong and Hong [16] reviewed 13 articles and determined that professional players had a higher injury rate than adolescent players during competitions, although Junge and Dvorak [10] reported a higher incidence among youth soccer players than among adults.

FIFA founded the FIFA Medical Assessment and Research Centre (F-MARC), which established criteria for epidemiological studies to investigate the characteristics of soccer injuries and to prevent injuries and illnesses in athletes [17,18]. The International Olympic Committee (IOC) have been collecting data on injuries of athletes at Olympic Games since the Beijing 2008 Olympic Games using an injury surveillance system (ISS) [19,20,21]. Based on the results of the IOC ISS, the injury prevention program “Get-Set” was developed and expanded. Because the time lost as a result of injuries in youth has a negative effect on soccer skills and performance, injury prevention and management are crucial. A previous study [22] proposed the development of the Translating Research into Injury Prevention Practice (TRIPP) framework, which applies the results of research studies to the implementation of injury prevention programs.

According to the TRIPP framework, there is a need to conduct an epidemiological study on youth soccer players to establish injury prevention strategies that are based on the phenomena of youth soccer injuries in Korea. However, although many researchers have investigated the physical performance of players [23,24], leadership of coaches [25], training systems for youth soccer players [26], and video analysis of injuries of professional soccer players [27] in Korea, few epidemiological studies have been conducted on Korean youth soccer players. Although investigating the phenomena of soccer injuries among both sexes is important, only 15 teams and 343 players play in Korean female U-15 soccer nationwide [3,4]. Given the proportion of youth soccer players of both sexes, focusing on the epidemiology of male soccer players first may be more beneficial to develop injury prevention strategies. Therefore, the goal of this study was to analyze the injury profiles and the severity of injury occurring during Korean male youth soccer training and matches.

## 2. Methods

### 2.1. Participants

Among 7838 Korean male U-15 soccer players from 235 teams, we randomly selected 24 teams, which accounted for approximately 10% of the total teams, and requested them to participate in this investigation. A total of 681 male U-15 soccer players from 22 teams accepted our request and agreed to participate. All selected players and teams were officially registered with KFA. Demographic characteristics of all participants are shown in Table 1.

### 2.2. Injury Report Forms and Data Collection

To conduct this retrospective study, eight investigators were trained about exact definitions and procedure of measurement, visited 22 selected teams, and instructed all participants to fill out the questionnaire about injuries diagnosed by medical doctors during the 2019 season. The questionnaire comprised questions on demographic information (e.g., age, career, height, weight, position, education on injury prevention regardless of type, and dominant leg), training condition (e.g., warm-up duration, training exposure, and field type), and information about injuries (e.g., location, occurrence, severity, type, cause, recurrent injury, surgery, days to return to full activities, and treatment expenses) based on the F-MARC consensus statement [18] and the IOC ISS [28]. The injury report form is shown in Supplementary Materials Questionnaire S1. Collected data were stored in an online database system using the IOC ISS. Information on soccer injuries from January 2019 to November 2019 was collected in December 2019.

#### 2.2.1. Definition of an Injury

An injury was defined as a physical complaint reported by a player experienced during a soccer match or training and included the following two factors: (1) a “medical attention” injury was defined as an injury that required a player to receive medical attention and (2) a “time loss” injury was considered an injury that rendered a player unable to participate in full training or a match [18].

#### 2.2.2. Definition of a Recurrent Injury

A recurrent injury was defined as an injury that occurred at the same site of the same type after a player’s full participation in a match or training after the index injury [18].

#### 2.2.3. Definition of a Match and Training

Training was defined as team-based and individual physical activities under the supervision of the team’s coaching or fitness staff. A match was considered a play between teams from different clubs irrespective of a friendly or official match [18].

#### 2.2.4. Injury Severity

Injury severity was graded according to the number of absences from full soccer activities such as team training or a match after injury diagnosed by medical doctors: (1) “slight” injury indicating 0 days lost, (2) “minimal” injury indicating 1–3 days lost, (3) “mild” injury indicating 4–7 days lost, (4) “moderate” injury indicating 8–28 days lost, and (5) “severe” injury indicating >28 days lost [18].

#### 2.2.5. Injury Classification

An injury was classified based on the IOC ISS [28]. The questionnaire included questions on information such as location, type, and cause of injury.

### 2.3. Statistical Analysis

All data obtained from the questionnaires were analyzed using IBM SPSS software version 25.0 (IBM Corp., Armonk, NY, USA). For each category, we calculated the absolute and relative frequency or mean and standard deviation. The injury rate was derived using the following formula: [(number of total injuries/number of total players) × 100] and/or [(number of injured players of specific position/number of total players of specific position) × 100].

## 3. Results

### 3.1. Demographic Characteristics

A total of 410 injuries were reported by 681 players aged 12–15 years during the 2019 season (injury rate: 60.2%). The most common player position was defender, which accounted for 33.0% (*n* = 225) of all positions. Regarding dominant legs, the right leg was dominant in 78.1% (*n* = 532) and the left leg and both legs in 12.9% (*n* = 88). Only 26% (*n* = 177) of all players had received education on injury prevention, with approximately 1.6 sessions per year (Table 1).

### 3.2. Training Conditions

The average warm-up duration in U-15 youth soccer was 18 min. Youth soccer players participated in training for approximately 117.6 min per session, 1.6 ± 0.77 sessions per day, 5.3 days per week, and 11.6 months per year. The most common type of training field was artificial ground (97.4%, *n* = 663; Table 2).

### 3.3. Characteristics of Injuries

As shown in Table 3, 56.3% (*n* = 231) of reported injuries occurred during training and the remaining during a match (43.7%, *n* = 179). Regarding severity, injuries were most commonly moderate (29.0%, *n* = 119), followed by severe (23.0%, *n* = 94) and mild (20.0%, *n* = 82). More than half of the defenders suffered an injury (54.7%, *n* = 123). The recurrent injury rate and surgery rate were 35.1% (*n* = 144) and 4.4% (*n* = 18), respectively. The average number of days before return to full activities was reported to be 23, and players paid approximately 130 USD per injury.

### 3.4. Locations of Injuries

The most common location of injuries among youth soccer players was the lower extremity (76.8%, *n* = 315), followed by the upper extremity (13.7%, *n* = 56) and the head and trunk (9.5%, *n* = 39). The top five injured body locations were the ankle (26.6%, *n* = 109), knee (14.1%, *n* = 58), thigh (12.4%, *n* = 51), foot/toe (11.0%, *n* = 45), and wrist (5.6%, *n* = 23; Table 4).

### 3.5. Types of Injuries

The most common type of injury was ligament sprain (21.0%, *n* = 86), followed by contusion (15.6%, *n* = 64), fracture (13.9%, *n* = 57), muscle strain (12.4%, *n* = 51), and ligament rupture (10.7%, *n* = 44; Table 5).

### 3.6. Causes of Injuries

As shown in Table 6, the most common cause of injuries was contact with other players (44.9%, *n* = 185). In addition, noncontact (22.2%, *n* = 87) and overuse (28.8%, *n* = 110) constituted a large portion of injuries.

## 4. Discussion

This retrospective epidemiological study was conducted to identify injury profiles of Korean male youth soccer players. Based on the data collected on injuries of Korean male youth soccer players, we identified that the most frequent type of injury was a new ankle injury of moderate severity with ligament sprain as a result of contact with other players during training. Medical staff members including clinicians, physiotherapists, nurses, and athletic trainers may need to consider these results when managing youth soccer players. The results of this study indicate that most youth soccer players in Korea play soccer on artificial grounds. According to a previous study [29], the incidence of injury on artificial grounds was higher than that on soft grounds, regardless of gender, training type, or match type. Moreover, previous studies found that among all positions, defenders have the highest incidence of injury [14,30]. The results of this study are similar to those of previous studies [14,30]. The most common position in Korean youth soccer is that of defenders, who are also most frequently injured in Korean youth soccer. This may imply that many injuries occur in Korean youth soccer players because these players play soccer on artificial grounds and most players are defenders. Furthermore, most Korean youth soccer players may have a high risk of injuries because only 26% of these players have received education on injury prevention. To the best of our knowledge, injury prevention programs in Korea are usually delivered through lectures but not through practical training and they usually focus on a specific population such as coaching staff. There are few chances of youth soccer players attending such injury prevention education programs. Therefore, we suggest (1) developing injury prevention educations involving practical and need-based training and (2) providing injury prevention education to youth soccer players to reduce injury rate and increase knowledge on injury management.

The recurrent injury rate in this study was 35.1% (*n* = 144) of all injuries occurred repeatedly. This figure is higher than that reported in a previous study, which found a recurrent injury rate of 15.1% in English youth soccer [31]. In Korean youth soccer, 29% (*n* = 119) of the total injuries were reported to be “moderate,” indicating an absence from soccer activities for 1–4 weeks. These results are similar to those of previous studies [30,31]. We calculated the number of days to return to full activities and found that 23 days were spent on rehabilitation on average. The average cost of injury treatment for youth soccer players was approximately 130 USD, and the surgery rate among all injuries was reported as 4.4%. Therefore, we suggest that the development of injury prevention programs based on the results of the IOC ISS for Korean youth soccer may be helpful in reducing both the cost of injury treatment and time lost from soccer activities regardless of age, sex, and play levels.

Many studies have reported the body location of soccer injuries [1,6,7,9,10,11,12,13,14,15,16,30,31]. Most injuries typically occurred at the lower extremity such as the ankle, knee joints, and thigh muscles. Our findings also show that the most common body locations of soccer injuries are the ankle joint, knee joint, and thigh muscles, with the lower extremity accounting for 76.8% of all injuries. These results are supported by those of previous research [1,6,7,9,10,11,12,13,14,15,16,30,31]. Because injuries of the lower extremity account for the majority of all injuries, F-MARC developed and suggested injury prevention programs such as “FIFA 11” and “FIFA 11 plus,” which focus on lower extremity function and its effects, as identified through previous studies [32,33]. Furthermore, the wrist, finger, and low back were found to be common sites of injury, which may indicate that protectors such as tapes and braces are applied to local body locations and their effects should be verified through prospective epidemiological studies.

Previous studies [29,30,31] reported that the most common types of injuries were muscle strains, ligamentous sprains, and contusions. In addition, fractures were among the top five most common soccer injuries in these studies [29,30,31]. Watson and Mjaanes [15] also found that fractures were very common soccer injuries in children and adolescents. Our findings were similar to those of previous studies [29,30,31]. Ligamentous sprains, contusions, fractures, muscle/tendon strains, and ligamentous ruptures were ranked among the top five injuries in this investigation. We determined the top three causes of injuries to be contact with other players, noncontact, and overuse with sudden onset. Based on these results, we recommend that experts in the field of sports medicine should consider the types and causes of injuries reported in this study when developing injury prevention programs. Although biomechanical studies in the laboratory setting have been conducted to identify mechanisms of injury and develop injury prevention programs [34,35,36], one limitation of a laboratory study design may be the controlled situation. Therefore, it is possible to analyze more exact mechanisms of injury using improved video recording and two-dimensional analytic techniques through a web-based ISS, which uses the video content analysis of a soccer match. In addition, exploration of the mechanisms and risk factors of an ankle sprain may be needed, given that in the present investigation, the most common body location and injury type were the ankle joint and ligament sprain, respectively. There are some limitations to this study. First, because injury information was collected retrospectively, the results may be affected by recall bias. Second, because participants of this study were only male, our results may not apply to female youth soccer players. Third, we were unable to report the incidence of injuries because we lack exact data on athlete-exposures (AEs) to training and matches. Fourth, we were unable to report more detailed information of injuries from the medical perspective because we used a simple epidemiological survey. Despite these limitations because our results were drawn from a large sample size (*n* = 681) and this study is a systematic report about injury profiles of youth soccer players, we expect that these results will be used to develop evidence-based injury prevention programs and education to reduce injury rate in Korean youth soccer.

## 5. Conclusions

Because of the study design, we were unable to determine the precise time of AEs to matches and training. Based on the collected data, we were unable to calculate the accurate incidence of injury using data on AEs. Furthermore, the use of questionnaires to acquire information on injuries of Korean youth soccer players may be associated with a recall bias. Therefore, we recommend collecting injury data prospectively to analyze the actual phenomena of injuries in Korean youth soccer. According to our results, Korean youth soccer train and play matches on artificial grounds, which may result in a higher risk of injury. Furthermore, the results of this study showed that the most frequent type of injury was a new ankle injury of moderate severity with ligament sprain caused by contact with other players during training. In addition, we found a higher recurrent injury rate than that reported in a previous study. Researchers and coaching staff should consider these results as a key to prevent injuries in youth soccer players. Furthermore, because injury prevention education may help to decrease the injury rate by educating players on injury management, we suggest developing well-defined injury prevention education involving practical and need-based training.

## Figures and Tables

**Table 1:** Demographic characteristics of U-15 youth soccer players during the 2019 season.

Variable (*n* = 681)	Value
Age (years)	13.6 ± 1.01
Height (cm)	166.66 ± 10.04
Weight (kg)	55.61 ± 10.12
Career (year)	4.02 ± 1.99
Position	
Attacker	209 (30.7)
Midfielder	182 (26.8)
Defender	225 (33.0)
Goalkeeper	54 (7.9)
More than two answers	10 (1.5)
No answer	1 (0.1)
Dominant leg	
Right	532 (78.1)
Left	88 (12.9)
Both legs	54 (7.9)
No answer	7 (1.0)
Received education on injury prevention	
Yes	177 (26.0)
No	499 (73.3)
No answer	5 (0.7)
Number of sessions attended for injury prevention per year	1.57 ± 1.11

Values are expressed as mean ± standard deviation or number (% of total injuries).

**Table 2:** Information about training conditions in U-15 youth soccer during the 2019 season.

Variable (*n* = 681)	Value
Warm-up duration (minutes)	18.11 ± 11.57
Training exposure	
Training duration per session (minutes)	117.64 ± 24.73
Training session per day (sessions)	1.59 ± 0.77
Training day per week (days)	5.28 ± 0.74
Training month per year (months)	11.55 ± 1.09
Type of training field	
Artificial ground	663 (97.4)
Soft ground	0 (0)
Hard ground	2 (0.3)
More than two answers	1 (0.1)
No answer	15 (2.2)

Values are expressed as mean ± standard deviation or number (% of total injuries).

**Table 3:** Characteristics of injuries in U-15 youth soccer during the 2019 season.

Variable (*n* = 410)	Value
Time of occurrence	
Training	231 (56.3)
Match	179 (43.7)
Severity	
Slight	35 (8.5)
Minimal	80 (19.5)
Mild	82 (20.0)
Moderate	119 (29.0)
Severe	94 (23.0)
Injury rate on position ^1^	
Attacker	95 (45.5)
Midfielder	97 (53.3)
Defender	123 (54.7)
Goalkeeper	25 (46.3)
Recurrent injury rate	144 (35.1)
Surgery rate	18 (4.4)
Days to return to full activities (days)	22.58 ± 35.95
Expenses for treatment (USD)	127.07 ± 519.65

Values are expressed as number (% of total injuries) or mean ± standard deviation. USD, US dollar. ^1^ Number (% of total players of each position).

**Table 4:** Location of injuries in U-15 youth soccer during the 2019 season.

Body Location (*n* = 410)	Number of Injuries (% of Total Injuries)
Lower Extremity	315 (76.8)
Ankle	109 (26.6)
Knee	58 (14.1)
Thigh	51 (12.4)
Foot/toe	45 (11.0)
Lower leg	18 (4.4)
Groin	14 (3.4)
Achilles tendon	14 (3.4)
Hip	6 (1.5)
Upper Extremity	56 (13.7)
Wrist	23 (5.6)
Finger	16 (3.9)
Shoulder/clavicle	9 (2.2)
Elbow	4 (1.0)
Hand	2 (0.5)
Thumb	2 (0.5)
Head and Trunk	39 (9.5)
Low back	17 (4.1)
Pelvis	12 (2.9)
Face	4 (1.0)
Ribs	3 (0.7)
Head	1 (0.2)
Cervical spine	1 (0.2)
Upper back	1 (0.2)

**Table 5:** Types of injury in U-15 youth soccer during the 2019 season.

Type of Injury (*n* = 410)	Number of Injuries (% of Total Injuries)
Ligament sprain	86 (21.0)
Contusion	64 (15.6)
Fracture	57 (13.9)
Muscle strain	51 (12.4)
Ligament rupture	44 (10.7)
Tendinitis/Tendinopathy	27 (6.6)
Stress fracture	18 (4.4)
Fasciitis	8 (2.0)
Subluxation/Dislocation	7 (1.7)
Muscle cramp	7 (1.7)
Osteoarthritis/Synovitis/Bursitis	5 (1.2)
Laceration/Abrasion	5 (1.2)
Tendon rupture	4 (1.0)
Lesion of meniscus/Cartilage	4 (1.0)
Impingement syndrome	4 (1.0)
Other bone injury	2 (0.5)
Nerve/Spinal cord injury	2 (0.5)
Other	15 (3.7)

**Table 6:** Causes of injury in U-15 youth soccer during the 2019 season.

Cause of Injury (*n* = 410)	Number of Injuries (% of Total Injuries)
Contact: other players	143 (34.9)
Noncontact	91 (22.2)
Overuse (sudden onset)	62 (15.1)
Overuse (gradual onset)	56 (13.7)
Contact: moving object	27 (6.6)
Contact: stagnant object	9 (2.2)
Contact: other people	5 (1.2)
Condition of field	4 (1.0)
Equipment failure	2 (0.5)
Other	11 (2.7)

## Supplementary Materials

The following are available online at https://www.mdpi.com/1660-4601/17/14/5125/s1, Questionnaire S1: Injury report form (youth soccer players) by the injury surveillance system collaboration between the IOC Research Centre Korea and K LEAGUE.
