# Supplementary material for: Injury Profiles in Korean Youth Soccer
Source: Int J Environ Res Public Health. 2020 Jul 16;17(14):5125. doi: 10.3390/ijerph17145125 (PMC7400017; doi:10.3390/ijerph17145125)
Supplement: Supplementary file 1 [file ijerph-17-05125-s001.pdf]

Questionnaire S1

IOC Research Centre
For Prevention of Injury and
Protection of Athlete Health
KOREA

K LEAGUE

Injury Report Form (Youth Soccer Players) by the Injury Surveillance System

Collaboration Between the IOC Research Centre KOREA and K LEAGUE

<Basic Information>

1. Age: __________ years

2. Position

□ Forward □ Midfielder □ Defender □ Goalkeeper

3. Athletic Career: __________years

4. Dominant Leg

□ Right □ Left □ Both

5. Height: __________cm

6. Weight: __________kg

7. Information About the Training Condition

Contents	Values
Duration of each training session	__________ minute
Number of training sessions held per day	__________ session
Number of training days per week	__________ day
Number of training months per year	__________ month
Type of training ground	□ Artificial turf □ Natural turf □ Hard ground

8. Average Duration of Warm-up: __________ minutes

9. Education Experience for Prevention of Injuries

□ Yes □ No

10. Number of Times Education for Injury Prevention was Received: __________ times

☞ **Go to the next page**

<Injury Information>

Please provide the information in the following table based on the criteria listed on injury information in the subsequent pages:

Contents	Injury 1	Injury 2	Injury 3	Injury 4
1. Date of injury				
2. Time that the injury occurred				
3. Days taken to return to full team training				
4. Bodily location of the injury				
5. Type of injury				
6. Cause of injury				
7. Recurrent injury				
8. Number of months elapsed from the index injury to the recurrent injury				
9. Experience of surgery caused by the injury				
10. Cost of treatment caused by the injury				

Contents	Injury 5	Injury 6	Injury 7	Injury 8
1. Date of injury				
2. Time that the injury occurred				
3. Days taken to return to full team training				
4. Bodily location of the injury				
5. Type of injury				
6. Cause of injury				
7.Recurrent injury				
8. Number of months elapsed from the index injury to the recurrent injury				
9. Experience of surgery caused by the injury				
10. Cost of treatment caused by the injury				

<Definition of injury information>

> ※ **Definition of injury**
>
> A soccer injury is considered as **any physical complaint** reported **by a player that occurred during a football match or a training session**, regardless of the "medical attention" or "time loss" from soccer activities.
> A "medical attention" injury is defined as an injury that requires a player to receive medical attention.
> A "time loss" injury is defined as an injury that results in a player being unable to fully engage in soccer training or a match play.

1. Date of injury

Please give **the date** when you were injured.

2. Time of injury occurrence

If you were injured during a training session, write "**training.**"

If you were injured during a match, write "**match.**"

* A match between the same team is not considered a "match" but rather "training."

3. Days elapsed before returning to full team training

Choose **the number of days** it took to recover from injury and participate in full team training.

(1) 0 day (2) 1–3 days (3) 4–7 days (4) 8–28 days (5) >28 days

4. Bodily location of injury

Choose the location on the body where you were injured.

Head and Trunk	Upper Extremity	Lower Extremity
(1) Face (eye, nose, ear, tooth, etc.)<br>(2) Head<br>(3) Neck<br>(4) Upper back<br>(5) Rib<br>(6) Low back<br>(7) Abdomen<br>(8) Pelvis<br>(9) Genitalia	(11) Shoulder, clavicle (anterior, posterior)<br>(12) Upper arm (anterior, posterior, medial, lateral)<br>(13) Elbow (medial, lateral)<br>(14) Forearm (anterior, posterior)<br>(15) Wrist (medial, lateral)<br>(16) Hand<br>(17) Finger<br>(18) Thumb	(21) Hip<br>(22) Groin<br>(23) Thigh (anterior, posterior, medial, lateral)<br>(24) Knee (medial, lateral)<br>(25) Lower leg (anterior, posterior, medial, lateral)<br>(26) Achilles tendon<br>(27) Ankle (anterior, medial, lateral)<br>(28) Foot, toe

5. Type of injury

Choose **the type of injury** you experienced.

(1) Contusion<br>(2) Fracture<br>(3) Stress fracture (overuse)<br>(4) Other bone injury<br>(5) Subluxation, dislocation<br>(6) Tendon rupture<br>(7) Ligament rupture<br>(8) Muscle strain<br>(9) Sprain (ligament, joint capsule)<br>(10) Osteochondral or meniscal lesion	(11) Contusion<br>(12) Tendinopathy<br>(13) Osteoarthritis, tenosynovitis, bursitis<br>(14) Fasciitis<br>(15) Impingement syndrome<br>(16) Abrasion<br>(17) Dental injury<br>(18) Nerve or spinal cord injury<br>(19) Muscle cramps<br>(20) Other

6. Cause of injury

Choose **the cause of injury** you experienced.

(1) Overuse (gradual onset)<br>(2) Overuse (sudden onset)<br>(3) Noncontact trauma	(11) Contact: another player<br>(12) Contact: another person<br>(13) Contact: moving object<br>(14) Contact: stagnant object	(21) Ground condition<br>(22) Weather<br>(23) Equipment failure<br>(24) Other

7. Recurrent injury

If you were injured at the same body location with the same type of injury again, write "**O**".

If you were injured at the body location first, write "**X**".

※ Recurrent injury is defined as an injury that occurred at **the same body location** with **the same type of injury** as **the index injury** suffered previously.

8. Months elapsed from the index injury to recurrent injury

Write **the number of months** that elapsed between the index injury and the recurrent injury.

9. Experience of surgery due to the injury

If you underwent surgery for the injury, write "**O**".

If you did not undergo surgery for the injury, write "**X**".

10. Expenses for treatment due to the injury

Write **the expenses** that resulted from the injury (e.g., diagnosis, treatment, surgery, etc.)
